# EBV-Encoded MicroRNA-BART17-3p Targets DDX3X and Promotes EBV Infection in EBV-Associated T/Natural Killer–Cell Lymphoproliferative Diseases

**DOI:** 10.1093/ofid/ofad516

**Published:** 2023-10-20

**Authors:** Jin Jin, Ting Sun, Man Zhang, Jiali Cheng, Jia Gu, Liang Huang, Min Xiao, Jianfeng Zhou, Hui Luo

**Affiliations:** Department of Hematology, Tongji Hospital, Tongji Medical College, Huazhong University of Science and Technology, Wuhan, Hubei, China; Immunotherapy Research Center for Hematologic Diseases of Hubei Province, Wuhan, Hubei, China; Department of Hematology, Renmin Hospital of Wuhan University, Wuhan, Hubei, China; Department of Hematology, Tongji Hospital, Tongji Medical College, Huazhong University of Science and Technology, Wuhan, Hubei, China; Immunotherapy Research Center for Hematologic Diseases of Hubei Province, Wuhan, Hubei, China; Department of Hematology, Cancer Hospital of Harbin Medical University, Harbin, Heilongjiang, China; Department of Hematology, Tongji Hospital, Tongji Medical College, Huazhong University of Science and Technology, Wuhan, Hubei, China; Immunotherapy Research Center for Hematologic Diseases of Hubei Province, Wuhan, Hubei, China; Department of Hematology, Tongji Hospital, Tongji Medical College, Huazhong University of Science and Technology, Wuhan, Hubei, China; Immunotherapy Research Center for Hematologic Diseases of Hubei Province, Wuhan, Hubei, China; Department of Hematology, Tongji Hospital, Tongji Medical College, Huazhong University of Science and Technology, Wuhan, Hubei, China; Immunotherapy Research Center for Hematologic Diseases of Hubei Province, Wuhan, Hubei, China; Department of Hematology, Tongji Hospital, Tongji Medical College, Huazhong University of Science and Technology, Wuhan, Hubei, China; Immunotherapy Research Center for Hematologic Diseases of Hubei Province, Wuhan, Hubei, China; Department of Hematology, Tongji Hospital, Tongji Medical College, Huazhong University of Science and Technology, Wuhan, Hubei, China; Immunotherapy Research Center for Hematologic Diseases of Hubei Province, Wuhan, Hubei, China; National Clinical Research Center for Hematologic Diseases, the First Affiliated Hospital of Soochow University, Suzhou, China; Department of Hematology, Tongji Hospital, Tongji Medical College, Huazhong University of Science and Technology, Wuhan, Hubei, China; Immunotherapy Research Center for Hematologic Diseases of Hubei Province, Wuhan, Hubei, China

**Keywords:** DDX3X, EBV-associated T/NK-cell lymphoproliferative disorders, EBV-miR-BART17-3p, RIG-I–like receptor pathway

## Abstract

**Background:**

Epstein-Barr virus (EBV) persistently infects T/natural killer (NK) cells causing an array of refractory EBV-associated T/NK-cell lymphoproliferative disorders. EBV-encoded microRNAs are important regulators for EBV latent infection and tumorigenesis. However, the roles of most EBV microRNAs in EBV-infected T/NK cells remain poorly understood.

**Methods:**

On the basis of a search of the doRiNA database and the BiBiServ2-RNAhybrid website, we predicted that EBV-miR-BART17-3p targeted DDX3X, and we verified the hypothesis by dual-luciferase reporter assay and cell function experiments. In addition, we collected 50 EBV-positive T-, B-, and NK-cell samples from the peripheral blood of EBV-positive cases to examine the role of EBV-miR-BART17-3p in the disease.

**Results:**

We found that EBV-miR-BART17-3p directly targeted DDX3X and downregulated DDX3X expression. By analyzing EBV-positive cell samples from cell lines and patients, we found that EBV-miR-BART17-3p was highly expressed only in EBV-positive NK cells and that the overexpression was significantly related to high EBV loads in EBV-infected NK cells. Furthermore, we found that EBV-miR-BART17-3p downregulated the RIG-I–like receptor antiviral pathway and promoted the expression of EBV-encoded proteins in EBV-infected NK cells by targeting DDX3X.

**Conclusions:**

Our study showed that EBV-miR-BART17-3p was abundantly expressed in EBV-infected NK cells and inhibited the important antivirus immune responses of hosts by targeting DDX3X of the RIG-I–like receptor pathway. These findings could help us gain insights into the pathogenic mechanisms underlying EBV-associated T/NK-cell lymphoproliferative disorders and find the potential therapeutic target.

## Teaser

EBV-encoded microRNA-BART17-3p abundantly expressed in EBV-infected NK cells and inhibited host important anti-virus immune pathway by targeting DDX3X of RIG-I-like receptor pathway, promoting EBV infection in NK cells. our study revealed the underlying pathogenic mechanisms of EBV-associated T/NK-cell lymphoproliferative disorders.

Epstein-Barr virus (EBV) represents a double-stranded DNA virus that infects most of the human population worldwide [1]. Most individuals can clear the virus without experiencing any significant clinical manifestations. EBV exhibits a predilection of B cells and rarely infects T and natural killer (NK) cells [1]. However, persistent EBV infection in T/NK cells can cause a variety of EBV-associated T/NK-cell lymphoproliferative disorders (EBV-T/NK-LPDs), such as chronic active EBV infection and EBV-associated hemophagocytic lymphohistiocytosis, among others [2]. The main clinical manifestations of EBV-T/NK-LPDs include high fever, lymphadenopathy, hepatosplenomegaly, liver function damage, and pancytopenia [3]. While the disease is relatively rare, patients with EBV-T/NK-LPDs tend to have an unfavorable prognosis and respond poorly to traditional treatments, such as supportive care, antiviral therapy, and chemotherapy [3, 4]. EBV-T/NK-LPDs mainly inflict children and young adults. In terms of EBV-infected cells, EBV-T/NK-LPDs fall into 3 types: NK cell, T cell, and mixed T and NK cells. We recently published a study about a cohort of adult-onset patients with EBV-T/NK-LPDs, in which the dominant EBV-infected cells were of the NK-cell type. The median survival time in our EBV-T/NK-LPD cohort was 140 days (range, 26–3783 days) [5], and the prognosis was less favorable in adult patients than in their child counterparts [6]. The mechanisms by which EBV infection persists in T/NK cells remain unclear. Primary immunodeficiencies in cytotoxicity and activation pathways involving CD8+ T cells and NK cells were reported to be associated with a high risk of severe EBV infection and the development of EBV-LPDs [7]. However, the primary immunodeficiencies were not found in a substantial number of patients with EBV-T/NK-LPDs. A question presents itself: How could EBV prevent the host immunity from effectively eliminating EBV-infected T/NK cells and maintain infection in T/NK cells?

Multiple studies have shown that EBV uses certain strategies to prevent host immune cells from removing EBV-infected cells and that the regulation by EBV-encoded microRNAs (miRNAs) is an important one [8]. EBV-encoded miRNAs are abundantly expressed in EBV-infected cells due to their low immunogenicity, including 25 miRNA precursors and 44 mature miRNAs [9]. Of the mature miRNAs, 4 are encoded in the BHRF1 region (BamHI fragment H rightward open reading frame 1), and the remaining are in the BART region (BamHI A rightward transcript). EBV miRNAs could regulate the gene expression of viruses and hosts in a variety of biological processes, such as viral replication, latency maintenance, immune escape, tumorigenesis, and disease progression [10, 11]. In addition, several EBV miRNAs have been reported to serve as biomarkers for the diagnosis, prognosis, and therapeutic efficacy of malignant tumors [12]. However, the roles of most EBV miRNAs in EBV-infected T/NK cells remain unknown.

The RIG-I–like receptor (RLR) pathway is a vital host antivirus pathway and was rarely studied in EBV infection in the past. Our recent investigation revealed that somatic *DDX3X* mutations and/or germline *IFIH1* mutations in the RLR pathway were frequent in EBV-T/NK-LPDs and could promote EBV infection in NK cells, which suggested the important role of this antiviral immune pathway [5]. The RLR family consists of *RIG-1* (retinoic acid–inducible gene 1), *MDA5* (melanoma differentiation–associated gene 5; also *IFIH1*), and *LGP2* (laboratory of genetics and physiology 2). The interaction of RIG-I/MDA5 with MAVS (mitochondrial antiviral signaling) leads to recruitment of downstream signaling molecules, including TRAF 3/6 (TNF receptor–associated factor 3/6) and IKK family members (inhibitor of NF-κB kinase; IKKε, TBK1 [TANK-binding kinase 1], and IKKα/β). Then activation of IRF-3/7 (interferon regulatory factor 3/7) transcriptionally activates interferon and proinflammatory cytokines, thereby enhancing the antiviral immune responses [13, 14]. DDX3X, a member of the RNA helicase family, is implicated in several important events of the RLR pathway. DDX3X has been reported to be directly associated with MAVS and IKK-ε/TBK1, thus taking part in interferon regulatory factor activation. Moreover, when RIG-I levels fall below the threshold required to trigger downstream signals, DDX3X might directly sense the viral RNA and sensitize the RLR pathway [15]. Patients with *DDX3X*/*IFIH1* aberrations accounted for virtually half of the overall cases of EBV-T/NK-LPDs (41.1%) [5]. Apart from the host genetic defects, it is still unknown whether EBV utilizes other strategies to circumvent the vital host antivirus immune system.

In this study, for the first time, we discovered that EBV highly expressed the EBV miRNA (EBV-miR-BART17-3p) in EBV-infected NK cells to inhibit the host vital antivirus immune pathway (ie, the RLR pathway) and promoted virus survival in NK cells by targeting DDX3X.

## METHODS

### Cell Lines and Cell Culture

The following EBV-positive B- and NK-cell lines were used: Raji from Burkitt lymphoma, NCI-BL2009 from EBV-transformed B lymphoblast, KAI3 from chronic active EBV disease, NK92 from aggressive NK-cell leukemia, YT from T/NK leukemia, and NKYS and SNK-6 from NK/T-cell lymphoma. IMC-1 came from EBV-negative (EBV–) aggressive NK-cell leukemia. The Raji, NCI-BL2009, NK92, and HEK293T cell lines in this study were obtained from the American Type Culture Collection. The KAI3, IMC-1, YT, NKYS, and SNK-6 cell lines were gifts from Dr Kai Fu at the University of Nebraska Medical Center. All cell lines were authenticated by short tandem repeat analysis before use. The culture conditions are summarized in Supplementary Table 1.

### miRNA Transfection

The SE Cell Line 4D-NucleofectorTM X Kit L (Lonza) was used to transfect mimics and inhibitors of EBV-miR-BART17-3p into NKYS cells in accordance with the manufacturer's guidelines. For each transfection, 2 × 10^6^ cells were mixed with 100nM miRNA mimics, inhibitors, or negative controls (NCs) in 100 μL of transfection solution and electroporated by the Lonza 4D electroporator with electroporation program CA-137. The mimics for EBV-miR-BART17-3p (UGUAUGCCUGGUGUCCCCUUAGU), inhibitor (ACUAAGGGGACACCAGGCAUACA), and NCs (UUUGUACUACACAAAAGUACUG) were procured from RiboBio. The cells were harvested 48 hours after transfection for real-time polymerase chain reaction (PCR) and Western blot analysis.

### RNA Isolation and Quantitative Real-time PCR

Total RNA was extracted from either cultured cells or patient samples resuspended in Trizol reagent with the Direct-zol RNA MiniPrep Kit (ZYMO Research). Complementary DNA was synthesized from 1 μg of total RNA with reverse transcriptase M-MLV (Takara) by following the manufacturer's instructions. The relative expression levels of host human genes and EBV-miR-BART4-3p were analyzed by quantitative real-time PCR, carried out on a CFX96 real-time PCR detection system (Bio-Rad), with SYBR Green All-in-One qPCR Mix (Bio-Rad). The primer sequences were listed in Supplementary Table 2. The relative transcriptional expression levels were determined by using the cycle threshold (2^−ΔΔCt^) method. Specifically, we first calculated the ΔCt of the experimental and control groups by using the following formula: Ct (target gene) − Ct (internal reference gene). Then ΔΔCt was computed as follows: ΔCt (experimental group) − ΔCt (control group). Finally, 2^−ΔΔCt^ was used to obtain the fold change of the target gene in the experimental group relative to the NC.

In the cell experiments involving DDX3X overexpression, GAPDH served as the internal reference gene and the cell lines transfected with no-load lentivirus as NCs. For the comparison of EBV-miR-BART17-3p expression levels among cell lines, U6 acted as the internal reference gene and the expression of miRNA in NKYS cells as the relative control. For experiments with different patient samples, GAPDH was used as the internal reference gene for the comparison of DDX3X expression, and U6 was employed for the comparison of miRNA expression. Depending on the cell number, the NK-cell sample of patient 277 was used as the relative control to normalize the expression levels of EBV-miRNA in all patient samples. All cell experiments were performed 3 independent times, each time in triplicate wells during quantitative PCR. The experiments for patient samples included specimens from >3 patients in a group, and 3 quantitative PCR wells were used for each reaction run.

### Construction of DDX3X-Overexpressing YT and HEK293T Cell Lines

Ubi-MCS-3FLAG-CBh-gcGFP-IRES-puromycin vector was purchased from GENECHEM, and the *DDX3X* gene was inserted into this vector. Then, HEK-293 T cells were cotransfected with the empty or DDX3X-expressing vectors with psPAX2 and PMD2.G packaging plasmids to generate lentiviral particles. YT and HEK293T cell lines were transduced with concentrated lentivirus at a multiplicity of infection ranging from 30 to 50. GFP-positive cells were flow cytometrically sorted 5 days after infection. YT and HEK293T cell lines transfected with the no-load lentivirus were used as NCs.

### Western Blot Analysis

The cultured cells were harvested and lysed with radioimmunoprecipitation assay buffer (Servicebio) containing 1% protease inhibitor phenylmethanesulfonyl fluoride and 2% protease inhibitor cocktail (Servicebio). Total protein lysate was then separated by 10% sodium dodecyl sulfate–polyacrylamide gel electrophoresis and transferred onto nitrocellulose membranes. Following blockade with 5% nonfat dry milk, the membranes were incubated overnight at 4 °C with primary antibodies. Subsequently, the membranes were washed 3 times and incubated with horseradish peroxidase–linked secondary antibody (AntGene) at room temperature. Finally, the expression levels of targeted proteins were detected with a Pierce ECL Western blotting substrate system (Bio-Rad). Specific antibodies were employed for DDX3X (1:2000; NOVUS), Flag (1:2000; Santa Cruz Biotechnology), BZLF1 (1:500; Santa Cruz Biotechnology), LMP1 (1:200; Santa Cruz Biotechnology), and EBNA1 (1:1000; Santa Cruz Biotechnology), with β-actin (1:2000; AntGene) used as the control.

### Luciferase Reporter Assay

By using the hg19 database and selecting 44 EBV-encoded mature miRNAs as regulators in the doRiNA website (https://dorina.mdc-berlin.de/), 608 transcripts of 441 genes were obtained [16]. We found that EBV-miR-BART17-3p had a regulatory effect on 3 transcripts of DDX3X. To predict the binding site between EBV-miR-BART17-3p and 3′-UTR of DDX3X, the RNAhybrid website (https://bibiserv.cebitec.uni-bielefeld.de/rnahybrid/) was searched. The DDX3X wild type, mutant, and NC reporter plasmids were acquired from RiboBio. Cotransfection of 50 ng of the recombinant plasmids and 50 nM of EBV-miR-BART17-3p mimics or NCs was performed in the HEK293T cells (1 × 10^4^ cells/well). After 48 hours of transfection, cell lysates were collected, and luciferase activity was measured by the dual-luciferase reporter system (Promega).

### Patients and Sample Collection

Samples were collected from patients receiving EBV-sorting PCR examination for diagnosis purposes in Tongji Hospital, Wuhan, China. As previously described, for EBV-sorting PCR examination [17], B, T, and NK cells were isolated from peripheral blood mononuclear cells, and EBV DNA copies in sorted cells were detected by EBV nucleic acid real-time PCR kits (Da An Gene) by following the manufacturer's instructions. The patients were diagnosed against the diagnostic criteria of the World Health Organization classification of lymphoid neoplasms (revised in 2016). This study was carried out upon approval by the Medical Ethics Committee of Tongji Medical College, Huazhong University of Science and Technology (2019S949), and all patients provided informed consent in strict accordance with the Declaration of Helsinki.

### Statistical Analysis

All experiments were repeated 3 times, and the bar charts were used to show the mean and SD of all 3 independent quantitative PCR experiments. The images representative of 3 independent Western blot analyses were presented. For cell experiments, an unpaired *t* test was used to compare 2 groups and analysis of variance for comparison among multiple groups. Nonparametric tests were conducted to compare differences among patient samples. The correlation was assessed per the Spearman correlation coefficient. *P* < .05 was considered statistically significant. Statistical analysis was performed with Prism software (version 9.0.0; GraphPad), and figures were formatted with Adobe Illustrator CS6.

## RESULTS

### EBV miRNAs Might Regulate DDX3X Expression

**Figure 1.**
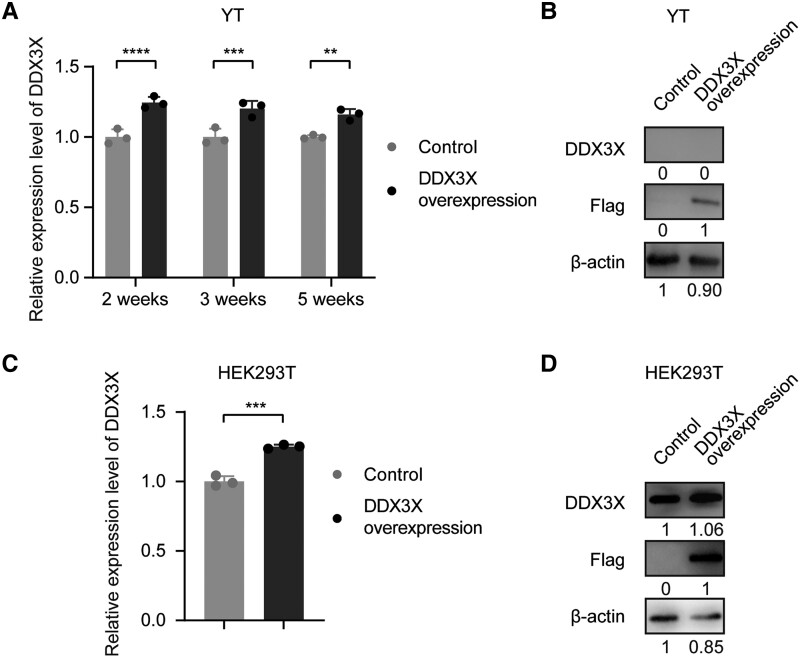
Epstein-Barr virus miRNAs might regulate DDX3X expression. *A*, Relative expression of DDX3X was measured in YT cells overexpressing DDX3X induced 2, 3, and 5 weeks after viral transfection. *B*, The protein expression levels of DDX3X and flag tag were detected in YT cells. *C*, The mRNA expression of DDX3X was measured in HEK293T cells. *D*, The protein expression levels of DDX3X and flag tag were detected in HEK293T cells. Statistical analysis was performed with an unpaired *t* test or analysis of variance. **P < .01. ***P < .001. ****P < .0001. Data are presented as mean ± SD.

In prior experiments, we observed that patients with EBV-associated lymphoproliferative diseases carrying genetic abnormalities in the RLR pathway had poorer overall survival [5]. Importantly, in patients with *DDX3X* aberrations in the RLR pathway, plasma and NK-cell EBV loads were elevated [5]. To investigate the mechanism underlying the elevation, we induced viral transfection–mediated overexpression of DDX3X in the EBV-positive (EBV+) NK-cell line YT. Surprisingly, the efficiency of DDX3X overexpression gradually dropped at the transcriptional level with the time of transfection, while DDX3X expression was practically undetectable at the protein level, although the flag tag linked to the DDX3X sequence could be detected (Figure 1*A* and 1*B*). We then transfected DDX3X into the EBV– cell line HEK293T and observed overexpression of DDX3X at the transcriptional and protein levels (Figure 1*C* and 1*D*).

Given that EBV is able to produce several miRNAs that function as viral regulators of host gene expression, we conducted a comprehensive analysis of all host human genes targeted by 44 EBV-encoded mature miRNAs in the doRiNA database (https://dorina.mdc-berlin.de/). Our analysis identified all possible genes or genetic fragments that could be targeted by EBV-encoded miRNAs, including 608 transcripts of 441 genes (Supplementary Data File 1). These results do not necessarily reflect the actual interaction and so warrant further experimental verification, including binding site prediction, luciferase reporter assay, and quantitative PCR. Of particular interest was the discovery that EBV-miR-BART17-3p exerted a regulatory effect on 3 transcripts of DDX3X, which might provide an explanation for the decreased DDX3X mRNA levels or even inappreciable levels of protein expression in the DDX3X overexpression experiments on EBV+ NK cells.

### EBV-miR-BART17-3p Directly Targeted DDX3X and Downregulated DDX3X Expression

**Figure 2.**
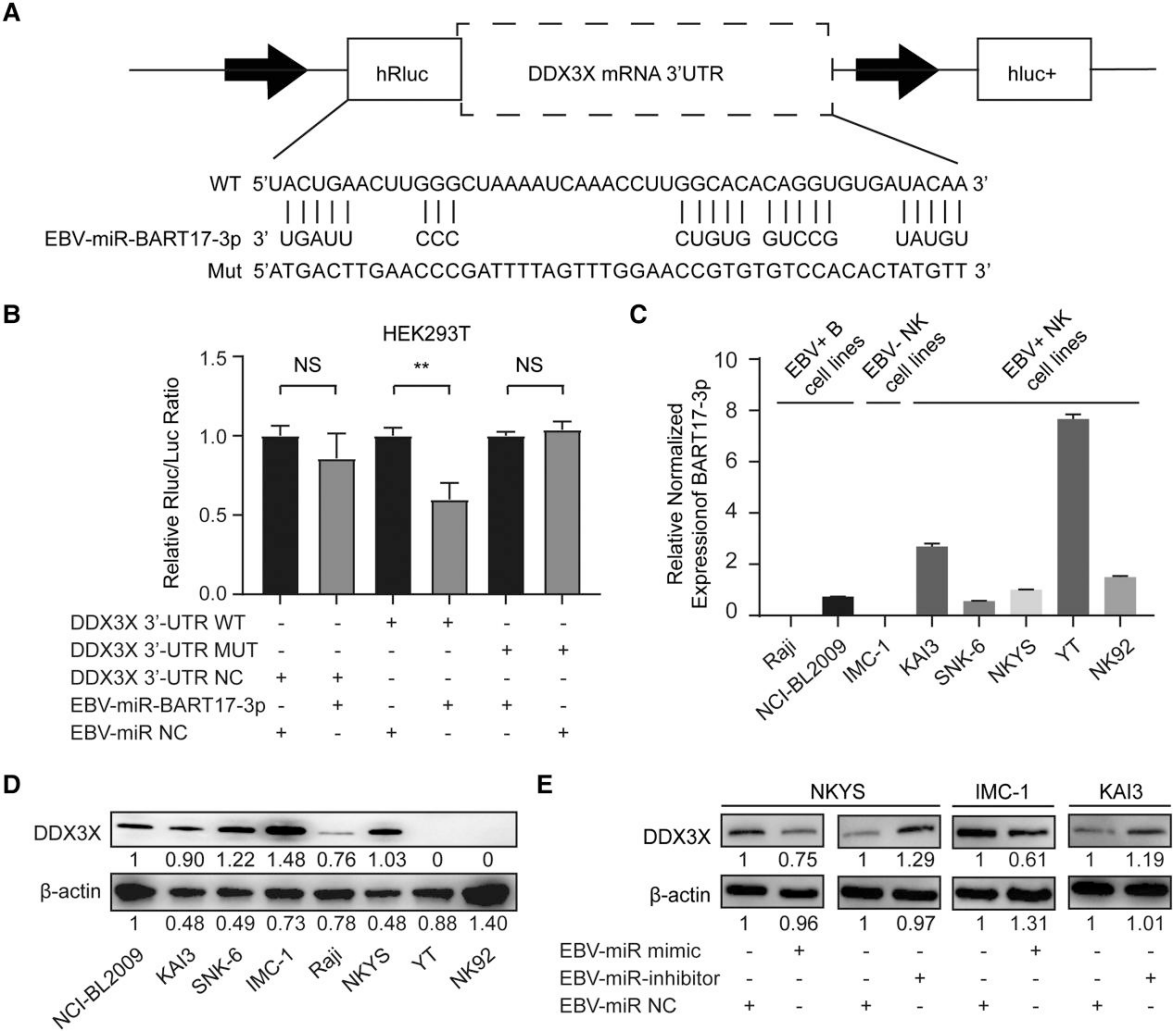
EBV-miR-BART17-3p directly targeted DDX3X. *A*, Matching results between the DDX3X 3′-UTR region and the EBV-miR-BART17-3p sequence, including the wild type and mutant type of the matching sequence. *B*, Negative control (DDX3X-NCs), wild type (DDX3X-WT), or mutated (DDX3X-MUT) plasmids were cotransfected with EBV-miR-BART17-3p mimics or miR-NCs into HEK293T cells. The luciferase activities were measured for 24 hours. Statistical analysis was performed with analysis of variance. NS, not significant (*P* > .05). **P < .01. *C*, The relative expression levels of EBV-miR-BART17-3p were measured in B-cell lines (NCI-BL2009 and Raji) and NK-cell lines (KAI3, SNK-6, IMC-1, NKYS, YT, and NK92). *D*, The protein expression level of DDX3X was detected in B-cell lines (NCI-BL2009 and Raji) and NK-cell lines (KAI3, SNK-6, IMC-1, NKYS, YT, and NK92). *E*, Comparison of DDX3X expression levels among NK-cell lines (NKYS, IMC-1, KAI3) transfected with EBV-miR-BART17-3p negative controls, inhibitors, or mimics. Data are presented as mean ± SD. EBV, Epstein-Barr virus; MUT, mutated; NC, negative control; NK, natural killer; WT, wild type.

To determine whether DDX3X is a direct target of EBV-miR-BART17-3p, we searched the BiBiServ2-RNAhybrid website to predict the binding site with the lowest free energy between EBV-miR-BART17-3p and the 3′-UTR of DDX3X, and the results suggested that the 2476-2523 region of the DDX3X coding sequence might be the miRNA binding site. Next, we constructed the DDX3X-MUT plasmid by mutating the aforementioned binding sites into their complementary bases to construct the DDX3X wild type and mutant reporter (Figure 2*A*). For dual-luciferase report analysis, NC plasmids (DDX3X-NCs), wild type plasmids (DDX3X-WT), or mutated plasmids (DDX3X-MUT) were cotransfected with EBV-miR-BART17-3p mimics or miR-NCs into HEK293T cells. As compared with miR-NCs, EBV-miR-BART17-3p significantly inhibited the activity of the firefly luciferase gene transfected with the wild type DDX3X plasmid (*P* = .0037), while the luciferase reporting activity of mutant DDX3X was not significantly altered (Figure 2*B*). These observations strongly suggested that DDX3X was a direct target of EBV-miR-BART17-3p.

To investigate the biological impact of EBV-miR-BART17-3p on DDX3X expression, we examined 2 EBV+ B-cell lines (NCI-BL2009 and Raji) and 6 NK-cell lines (SNK-6, NKYS, IMC-1, YT, NK92, and KAI3) for the expression of EBV-miR-BART17-3p and DDX3X (Figure 2*C* and 2*D*). We transfected EBV+ NKYS with EBV-miR-BART17-3p mimics and inhibitors, given its moderate levels of DDX3X and EBV-miR-BART17-3p expression. The results showed that transfection with EBV-miR-BART17-3p mimics for 48 hours led to a significant reduction of DDX3X at the protein level, whereas transfection with EBV-miR-BART17-3p inhibitors for 48 hours resulted in a significant increase of DDX3X (Figure 2*E*). We then verified these findings in other cell lines. EBV-miR-BART17-3p mimics were transfected into EBV– IMC-1 cell lines with high DDX3X expression, which also led to a significant reduction in DDX3X protein levels. Conversely, in EBV-positive KAI3 cell lines with low DDX3X and high miR-BART17-3p expression, the transfection of EBV-miR-BART17-3p inhibitors increased DDX3X expression (Figure 2*E*). These results showed that EBV-miR-BART17-3p mimics promoted DDX3X expression, while EBV-miR-BART17-3p inhibitors had opposite effects. Combined with the results of the luciferase reporter assay, we were led to conclude that EBV-miR-BART17-3p directly targeted DDX3X and could downregulate its expression in EBV+ cells.

### EBV-miR-BART17-3p Promoted EBV Infection in EBV+ NK Cells by Modulating DDX3X Expression

Previous studies have demonstrated that DDX3X facilitated EBV infection and promoted EBV-related gene expression in EBV+ NK cells by modulating the RLR pathway, thereby playing a pivotal role in the development and progression of EBV-T/NK-LPDs [5]. Our investigation on EBV+ cell lines revealed a higher expression of EBV-miR-BART17-3p in EBV+ NK-cell lines but a lower or undetectable expression in EBV+ B-cell lines (Figure 2*C*). Notably, the EBV+ NK-cell line (YT) had an exceptionally high expression of EBV-miR-BART17-3p, resulting in an inappreciable DDX3X expression (Figure 2*C* and 2*D*). These findings suggested that EBV-miR-BART17-3p might primarily function in EBV+ NK cells.

**Figure 3.**
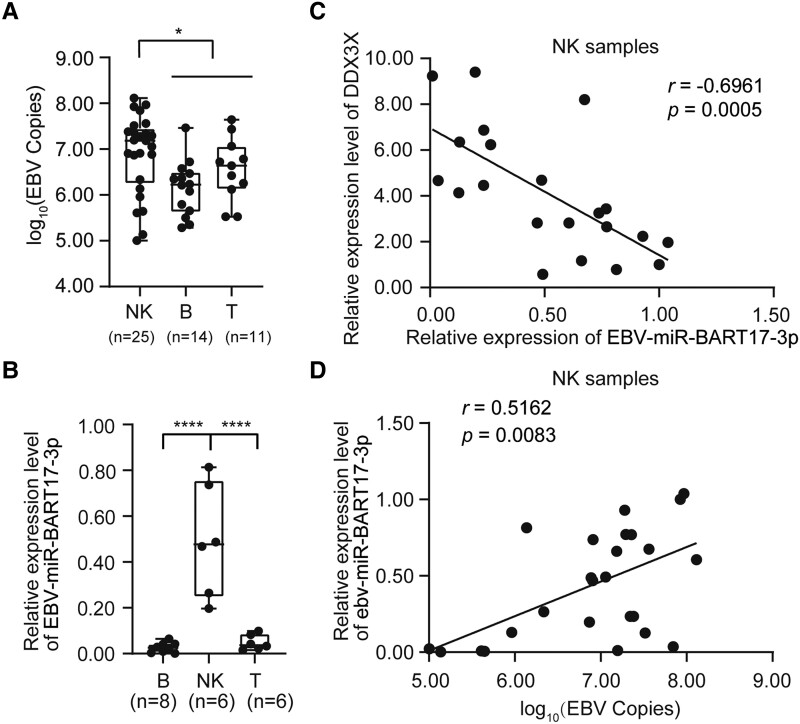
EBV-miR-BART17-3p promoted EBV infection in EBV + NK cells by modulating the expression of DDX3X. *A*, Comparison of EBV loads between NK-cell samples and T/B-cell samples from patients. *B*, Comparison of the relative expression levels of EBV-miR-BART17-3p in B-, NK-, and T-cell samples from patients with EBV loads between 10^6^ and 10^7^ copies/2 × 10^5^ cells. *C*, Analysis of the correlation between EBV-miR-BART17-3p expression and DDX3X levels in NK-cell samples (*r* = −0.6961, *P* = .0005). *D*, Analysis of the correlation between EBV-miR-BART17-3p expression and EBV loads in NK-cell samples (*r* = 0.5162, *P* = .0083). Statistical analysis was performed with the Mann-Whitney test. Correlation was evaluated in terms of the Spearman correlation coefficient. NS, not significant (*P* > .05). *P < .05. ****P < .0001. Data are presented as median (line), IQR (box), and range (error bars). EBV, Epstein-Barr virus; NK, natural killer.

To further investigate the role that EBV-miR-BART17-3p plays in EBV-associated diseases, we retrospectively collected 50 EBV-positive cell specimens from 42 patients undergoing EBV-sorting PCR examination at Wuhan Tongji Hospital. Their diseases included chronic EBV infection (n = 6), chronic active EBV infection (n = 26), EBV-associated hemophagocytic lymphohistiocytosis (n = 3), aggressive NK-cell leukemia (n = 7), and immune-related pancytopenia (n = 2), as well as several types of lymphoma: 2 cases of angioimmunoblastic lymphoma, 3 cases of NK/T-cell lymphoma, and 1 case of anaplastic large cell lymphoma. Among these 50 EBV-positive cell samples, 14 were B cells, 25 NK cells, and 11 T cells. The baseline characteristics of these patients are summarized in Supplementary Table 3. Of note, the EBV loads were significantly higher in NK-cell samples than in their T- and B-cell counterparts (*P* = .0116; Figure 3*A*). To rule out the effect of substantially different EBV loads on EBV miRNA expression, we used T-, B-, and NK-cell samples with EBV loads ranging between 10^6^ and 10^7^ copies/2 × 10^5^ cells for the comparison of EBV-miR-BART17-3p expression levels. Consistent with the results obtained from cell lines, EBV-miR-BART17-3p expression was significantly higher in EBV+ NK-cell samples than in EBV+ T- and B-cell samples (*P* < .0001; Figure 3*B*), indicating that EBV-miR-BART17-3p was principally expressed in EBV+ NK cells.

Moreover, we observed a significant negative correlation between the expression levels of EBV-miR-BART17-3p and DDX3X in NK-cell samples with EBV loads >5 × 10^5^ copies/2 × 10^5^ cells (*r* = −0.6961, *P* = .0005; Figure 3*C*), suggesting that the expression of DDX3X in EBV+ NK cells was regulated by EBV-miR-BART17-3p. To determine the effect of EBV-miR-BART17-3p on EBV infection in NK cells, we analyzed the correlation between the expression of EBV-miR-BART17-3p and EBV loads. The data revealed that the level of EBV-miR-BART17-3p bore a significant positive correlation with EBV loads in 25 EBV+ NK-cell samples (*r* = 0.5162, *P* = .0083; Figure 3*D*), which suggested that EBV-miR-BART17-3p potentially promoted EBV infection in these cells.

### EBV-miR-BART17-3p Downregulated the RLR Pathway and Promoted EBV Gene Expression

**Figure 4.**
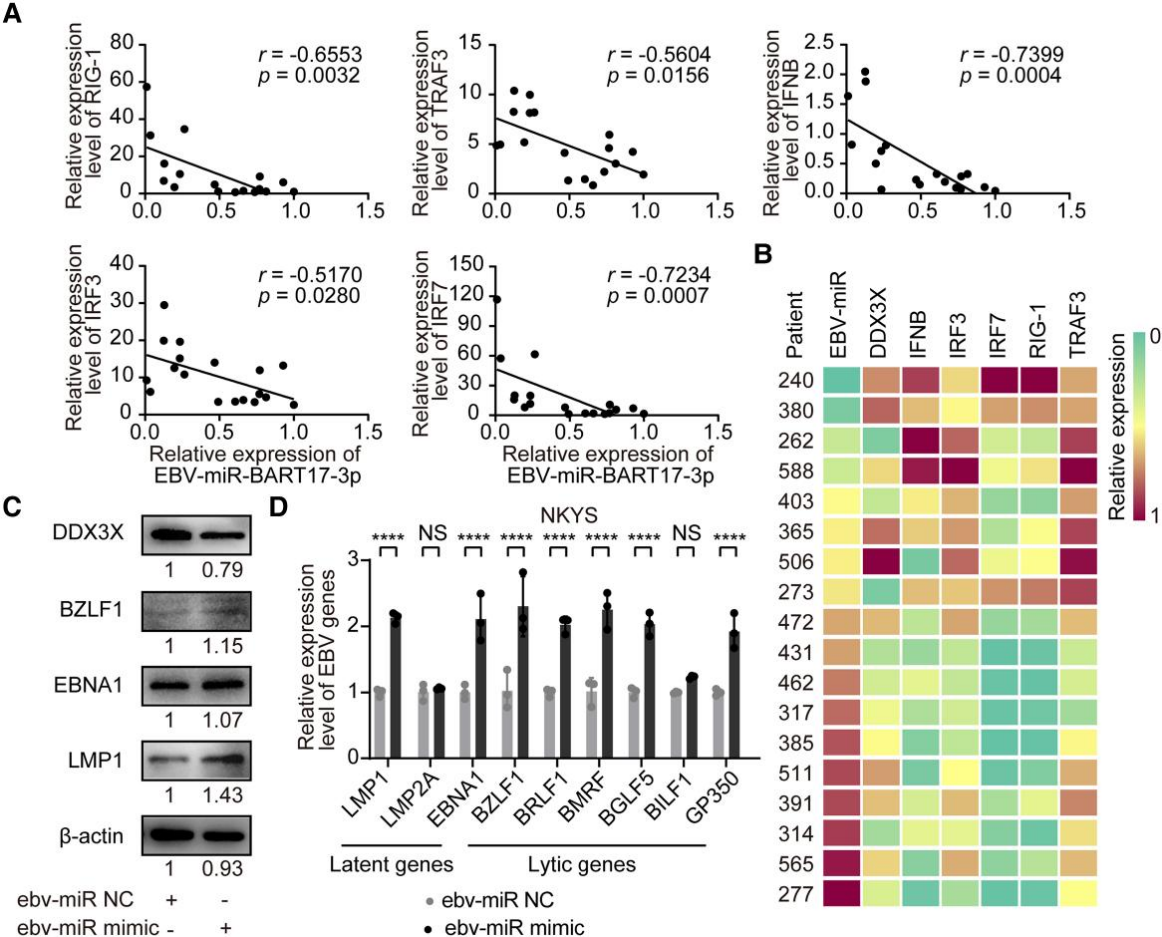
EBV-miR-BART17-3p downregulated the RLR pathway and promoted EBV gene expression. *A* and *B*, The expression levels of EBV-miR-BART17-3p and genes in the RLR pathway, including *IRF3*, *IRF7*, *IFNB*, *RIG-1*, and *TRAF3*, in 18 NK-cell samples from patients. *C*, The protein expression levels of DDX3X and representative EBV lytic/latent proteins (*BZLF1, EBNA1, LMP1*) were determined in NKYS cells transfected with EBV-miR-BART17-3p negative controls and mimics by Western blotting. *D*, The transcriptional levels of EBV latent genes (*LMP1*, *LMP2A*, *EBNA1*), EBV immediate early lytic genes (*BZLF1*, *BRLF1*), EBV early lytic genes (*BMRF*, *BGLF5*), and late lytic genes (*BILF1*, *GP350*) were determined in NKYS cells transfected with EBV-miR-BART17-3p negative controls and mimics by real-time polymerase chain reaction. Statistical analysis was performed with analysis of variance. Correlation was evaluated in terms of the Spearman correlation coefficient. NS, not significant (*P* > .05). ****P < .0001. Data are presented as mean ± SD. EBV, Epstein-Barr virus; NC, negative control; NK, natural killer; RLR, RIG-I–like receptor.

To further understand the impact of EBV-miR-BART17-3p on the RLR pathway via the DDX3X, we examined the expression levels of 6 downstream genes of the RLR pathway—*DDX3X*, *IRF3*, *IRF7*, *IFNB*, *RIG-1*, and *TRAF3*—in 18 NK patient samples, which were from 21 patients with EBV loads >5 × 10^5^ copies/2 × 10^5^ cells in NK cells. Among the 21 cases, 3 were excluded due to insufficient samples. The results revealed that EBV-miR-BART17-3p was significantly negatively correlated with the downstream genes of the RLR pathway, suggesting that the high level of EBV-miR-BART17-3p could downregulate the RLR pathway in EBV+ NK cells by targeting DDX3X (Figure 4*A* and 4*B*).

Prior research has demonstrated that the reduced DDX3X expression could upregulate EBV-related gene expression. To investigate the impact of EBV-miR-BART17-3p on EBV-related genes, we utilized electrotransfection to introduce EBV-miR-BART17-3p mimics and miR-NCs into NKYS cell lines. Our findings revealed that EBV-miR-BART17-3p resulted in a downregulation of DDX3X expression, leading to a substantial increase in a range of EBV genes, including latent genes (*LMP1*, *LMP2A*, *EBNA1*), immediate early lytic genes (*BZLF1*, *BRLF1*), early lytic genes (*BMRF*, *BGLF5*), and late lytic genes (*BILF1*, *GP350*) at the transcriptional level. We also observed an increase in protein expression of 3 critical EBV genes (*BZLF1*, *EBNA1*, *LMP1*; Figure 4*B* and 4*C*). Our findings suggested that EBV-miR-BART17-3p could promote the expression of EBV genes by downregulating DDX3X expression.

## DISCUSSION

In this study, we found that EBV-miR-BART17-3p directly targeted DDX3X and downregulated DDX3X expression. EBV-miR-BART17-3p was highly expressed exclusively in EBV-infected NK cells rather than in EBV-infected B or T cells, and its overexpression was significantly correlated with high EBV loads. In addition, the elevated expression of EBV-miR-BART17-3p could downregulate the RLR antiviral pathway and promote the expression of EBV-encoded proteins in EBV-infected NK cells. Thus, we were led to speculate that (1) EBV-miR-BART17-3p might be one of the most important factors mediating EBV immune invasion in NK cells and (2) DDX3X in the RLR pathway, targeted by EBV-miR-BART17-3p, might be pathogenetically implicated in the development of the NK-cell type of EBV-T/NK-LPDs.

**Figure 5.**
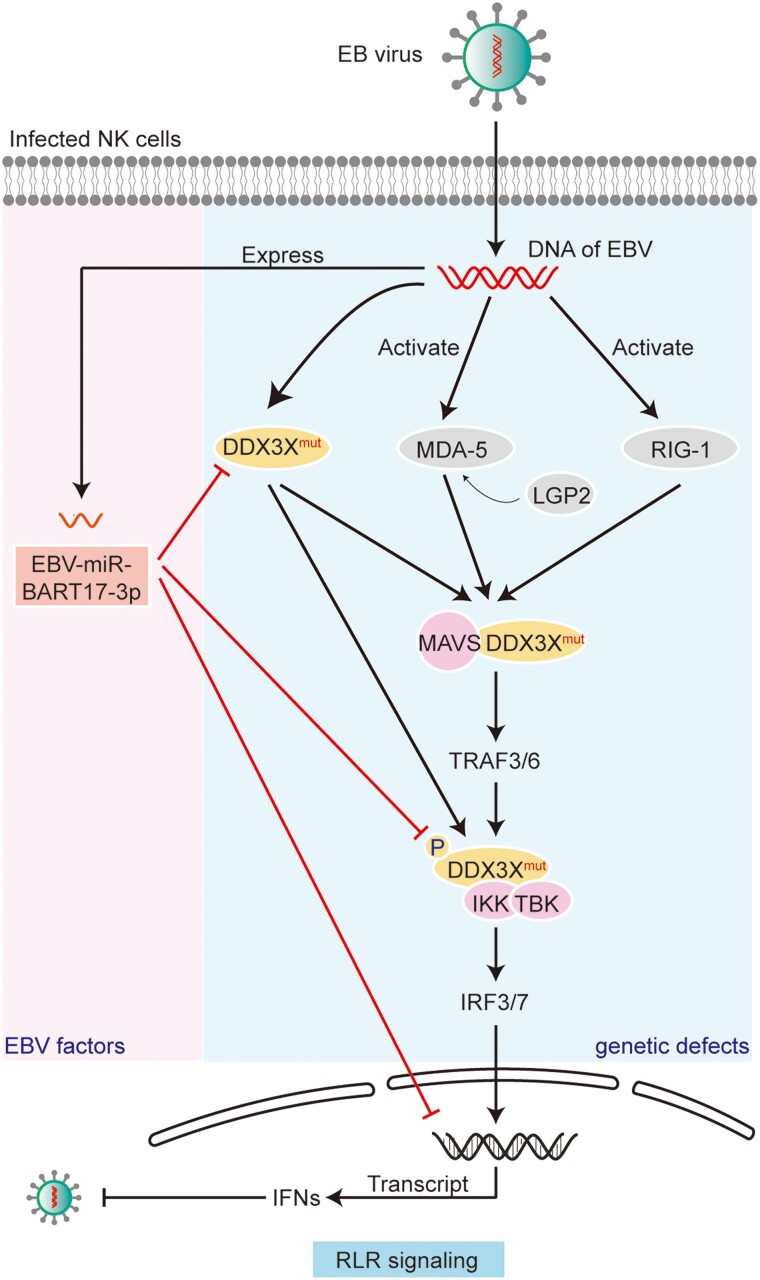
The proposed mechanism of persistent EBV infection in NK cells. Normally, RLR family members RIG-1 and MDA5, supported by LGP2, are able to sense the viral RNAs once NK cells are infected by EBV. After activation, RLRs interact with MAVS to recruit downstream signaling molecules (IKKε, TBK1, and IKKα/β) and activate IRF-3/7, which leads to IFN secretion, thereby enhancing the antiviral immune response. Defects in the host *DDX3X* gene negatively affect multiple molecules of RLR signaling (blue background), including its interactions with MAVS and TBK1, thus downregulating the secretion of IFNs. In addition, EBV can further inhibit the RLR antiviral pathway by expressing EBV-miR-BART17-3p targeting DDX3X (pink background). In conclusion, the genetic defects and viral factors in the RLR pathway might synergistically result in impaired immune function and persistent EBV infection in NK cells. EBV, Epstein-Barr virus; IFN, interferon; IKK, inhibitor of NF-κB kinase; IRF3/7, IFN regulatory factor 3/7; LGP2, laboratory of genetics and physiology 2; MAVS, mitochondrial antiviral signaling; MDA-5 (also IFIH1), melanoma differentiation–associated gene 5; NK, natural killer; RIG-1, retinoic acid–inducible gene 1; RLR, RIG-I–like receptors; TBK, TANK-binding kinase; TRAF3/6, TNF receptor–associated factor 3/6.

Our previous study found that >40% of patients with EBV-T/NK-LPDs had IFIH1 and DDX3X aberrations in the RLR pathways, which played a critical role in EBV-infected NK cells. The previous work focused only on the genetic defects of the RLR pathways (blue background in Figure 5) and did not examine other possible mechanisms involving the RLR pathways, such as the influence of the EBV virus itself [5]. In this study, we, for the first time, discovered that EBV-encoded miRNA-BART17-3p was richly expressed in EBV-T/NK-LPDs of the NK-cell type and revealed the mechanism by which the EBV-encoded miRNA regulated the host RLR pathway and promoted EBV infection in NK cells (pink background in Figure 5). On the strength of the results of the 2 studies, we believe that, mechanistically, the persistent EBV infection in T/NK cells was affected by host genetic features and the virus per se and that the RLR pathway was the pivotal target pathway. In EBV-associated diseases, the combined effect of genetics and virus factors is not uncommon, such as the activated JAK/STAT pathway being affected by the frequent pathway mutations and the EBV oncogenes such as *LMP1* [18]. Our research offered a good example of such combined effects by virus and host.

Our study also demonstrated that EBV-miR-BART17-3p might be one of the most important factors mediating the EBV immune invasion in NK cells. Moreover, such high expression of EBV-miR-BART17-3p was found only in EBV-infected NK cells, which targeted the RLR pathway, an important antivirus pathway. When compared with the EBV-encoded proteins, the EBV miRNAs have less immunogenicity [19]. Therefore, EBV miRNAs are not easily cleared by the host immune system and could be stably expressed in host cells. In fact, our data revealed a significant positive correlation between EBV-miR-BART17-3p levels and EBV DNA loads in EBV+ NK-cell samples (*r* = 0.5162, *P* = .0083; Figure 3*D*). Nevertheless, no correlation was found in 11 EBV+ T-cell samples (*r* = −0.3371, *P* = .3078; Supplementary Figure 1). Therefore, we were inclined to speculate that EBV-miR-BART17-3p might lead to higher EBV DNA loads in NK cells. In our study, EBV-miR-BART17-3p downregulated the RLR pathway and promoted EBV-related gene expression, which might contribute to persistent EBV infection in NK cells.

In summary, in this study, we presented evidence that DDX3X in the RLR pathway was a target of EBV-miR-BART17-3p. Although the RLR pathway was also affected by genetic mutations, no efficacious treatment but allo-HSCT is available for these patients with mutations. For EBV-T/NK-LPD in patient without mutations in the RLR pathway, EBV-miR-BART17-3p might be a potential therapeutic target.

## Supplementary Material

ofad516_Supplementary_Data

## Supplementary Data

Supplementary materials are available at *Open Forum Infectious Diseases* online. Consisting of data provided by the authors to benefit the reader, the posted materials are not copyedited and are the sole responsibility of the authors, so questions or comments should be addressed to the corresponding author.

## Notes

***Acknowledgments.*** The authors are indebted to all members and staff in the study team for their generous assistance and support.

***Author contributions.*** H. L. designed and supervised the study. H. L., J. G., M. X., and L. H. were responsible for diagnosis and provided the clinical data of patients. J. J., M. Z., T. S., and J. C. performed experiments. J. J. and H. L. wrote and revised the manuscript. J. Z. provided support for the project. All authors read and approved the final manuscript.

***Financial support*.** This work was supported by the National Natural Science Foundation of China (81830008 to J. Z.); a Translational Research Grant of the National Clinical Research Center for Hematologic Diseases of China (2020ZKZC03 to J. Z.); and the Cultivation Project of the Tongji Hospital Foundation (2022B18 to H. L.).

***Potential conflicts of interest*.** All authors: No reported conflicts.
