# Supplementary material for: EBV-Encoded MicroRNA-BART17-3p Targets DDX3X and Promotes EBV Infection in EBV-Associated T/Natural Killer–Cell Lymphoproliferative Diseases
Source: Open Forum Infect Dis. 2023 Oct 20;10(11):ofad516. doi: 10.1093/ofid/ofad516 (PMC10652706; doi:10.1093/ofid/ofad516)
Supplement: ofad516_Supplementary_Data [file ofad516_supplementary_data.zip › Supplemental Material.docx]

Supplement to ***Jin Jin, et al. EBV-encoded MicroRNA-BART17-3p Targets DDX3X and Promotes EBV Infection in EBV-associated T/NK-cell Lymphoproliferative Diseases***

This Supplemental Materials has been provided by the authors to give readers additional information about their work

**SUPPLEMENTARY MATERIALS**

**CONTENTS:**

**SUPPLEMENTARY TABLES** 3

Table S1. Culture conditions of all cell lines in this study 3

Table S2. Primers for quantitative RT-PCR 4

Table S3. The baseline characteristics of patients in this study 5

**SUPPLEMENTARY FIGURES** 8

Figure S1. Correlation analysis between EBV-miR expression and DDX3X levels 8

| **Table S1. Culture conditions of all cell lines in this study** | | | |
| --- | --- | --- | --- |
| Cell lines | Type | Source | Culture conditions |
| Raji | Burkitt lymphoma | ATCC | RPMI 1640 medium (Gibco, NY, USA) supplemented with 10% FBS (Gibco, NY, USA) |
| NCI-BL2009 | EBV-transformed B lymphoblast | ATCC | RPMI 1640 medium supplemented with 10% FBS |
| KAI3 | Chronic active EBV disease | A gift from Dr Kai Fu | RPMI 1640 medium containing 10% FBS and 100 U/ml recombinant human IL-2 (PeproTech, NJ, USA) |
| NK92 | Aggressive NK-cell leukemia | ATCC | RPMI 1640 medium containing 12.5% FBS, 12.5% horse serum, and 100 U/ml recombinant human IL-2 |
| IMC-1 | Aggressive NK-cell leukemia | A gift from Dr Kai Fu | RPMI 1640 medium containing 10% FBS and 100 U/ml recombinant human IL-2 |
| YT | T/NK leukemia | A gift from Dr Kai Fu | RPMI 1640 medium containing 10% FBS and 100 U/ml recombinant human IL-2 |
| NKYS | NK/T-cell lymphoma | A gift from Dr Kai Fu | RPMI 1640 medium containing 10% FBS and 100 U/ml recombinant human IL-2 |
| SNK-6 | NK/T-cell lymphoma | A gift from Dr Kai Fu | RPMI 1640 medium containing 10% FBS and 100 U/ml recombinant human IL-2 |
| HEK293T | Human embryonic kidney | ATCC | DMEM medium (Gibco, NY, USA) supplemented with 10% FBS |
| ATCC, American Type Culture Collection; FBS, fetal bovine serum | | | |

**SUPPLEMENTARY TABLES**

| **Table S2. Primers for quantitative RT-PCR** | | |
| --- | --- | --- |
| **Genes** | **Forward primer (5' to 3')** | **Reverse primer (5' to 3')** |
| DDX3X | GATGCTGGCTCGTGATTTCTTAG | TGTCTGATTCTTCCACCCAAACTA |
| RIG-1 | TGCGAATCAGATCCCAGTGTA | TGCCTGTAACTCTATACCCATGT |
| IFNB | ATGACCAACAAGTGTCTCCTCC | GGAATCCAAGCAAGTTGTAGCTC |
| TRAF3 | GCGTGTCAAGAGAGCATCGTT | GCAGATGTCCCAGCATTAACT |
| IRF3 | CACAGCAGGAGGATTTCGGAAT | TTATGTGGGTCGTGAGGGTCCT |
| IRF7 | CCCACGCTATACCATCTACCT | GATGTCGTCATAGAGGCTGTTG |
| P21 | TGTCCGTCAGAACCCATGC | AAAGTCGAAGTTCCATCGCTC |
| LMP1 | AATTTGCACGGACAGGCATT | AAGGCCAAAAGCTGCCAGAT |
| LMP2A | CCGTCACTCGGACTATCAAC | TGAGATGAGTCATCCCGTGGA |
| EBNA1 | GAGCGGGGAGATAATGTACA | TAAAAGATGGCCGGACAAGG |
| BZLF1 | TCCTCGTGTAAAACATCTGGTG | AATGCCGGGCCAAGTTTAAG |
| BRLF1 | AATTTACAGCCGGGAGTGT | AGCCCGTCTTCTTACCCTGT |
| BMRF | GAGGAACGAGCAGATGATTGG | TGCCCACTTCTGCAACGA |
| BGLF5 | TCAACAGATAGTCACCCTCCGA | TGACCCCATCTACCCATCCTA |
| BILF1 | GTATGGCGTTGGAGAAGACC | TAATCAGCAGGAGTACCAGACA |
| GP350 | GTCAGTACACCATCCAGAGCC | TTGGTAGACAGCCTTCGTATG |
| GAPDH | GACAGTCAGCCGCATCTTCT | TTAAAAGCAGCCCTGGTGAC |

| **Table S3. The baseline characteristics of patients in this study** | | | | | | |
| --- | --- | --- | --- | --- | --- | --- |
| **Sample No.** | **Patient No.** | **Cell type of samples** | **Diagnosis** | **EBV copies / 2x10^^5^ cells** | **miRNA relative expression** | **DDX3X relative expression** |
| 1 | 29 | T | Chronic Active EBV Infection | 1.33E+06 | 0.04172 |  |
| 2 | 84 | B | Chronic Active EBV Infection | 1.72E+06 | 0.00413 |  |
| 3 | 85 | B | Chronic EBV Infection | 2.26E+05 | 0.00126 |  |
| 4 | 85 | T | Chronic Active EBV Infection | 1.81E+06 | 0.02244 |  |
| 5 | 89 | B | Chronic EBV Infection | 4.51E+05 | 0.00086 |  |
| 6 | 118 | B | Chronic EBV Infection | 6.20E+05 | 0.00882 |  |
| 8 | 188 | T | Chronic Active EBV Infection | 1.16E+07 | 1.03861 | 1.96870 |
| 7 | 188 | NK | Chronic Active EBV Infection | 9.27E+07 | 0.00765 |  |
| 9 | 208 | T | Chronic Active EBV Infection | 3.37E+05 | 0.01296 |  |
| 10 | 220 | B | Chronic Active EBV Infection | 5.28E+06 | 0.04246 |  |
| 11 | 240 | NK | Aggressive Natural Killer Cell Leukemia | 1.57E+07 | 0.01042 | 9.23513 |
| 12 | 262 | NK | Chronic Active EBV Infection | 3.28E+07 | 0.12511 | 4.13638 |
| 14 | 273 | B | Chronic EBV Infection | 1.21E+06 | 0.26495 | 6.23672 |
| 13 | 273 | NK | Aggressive Natural Killer Cell Leukemia | 2.17E+06 | 0.00160 |  |
| 15 | 277 | NK | Aggressive Natural Killer Cell Leukemia | 8.44E+07 | 1.00000 | 1.00000 |
| 16 | 310 | T | Chronic Active EBV Infection | 2.64E+06 | 0.01570 |  |
| 17 | 314 | NK | EBV-associated Hemophagocytic Lymphohistiocytosis | 1.37E+06 | 0.81375 | 0.78526 |
| 18 | 317 | NK | Aggressive Natural Killer Cell Leukemia | 1.53E+07 | 0.66088 | 1.17173 |
| 19 | 342 | B | Immune-related Pancytopenia | 1.93E+05 | 0.00156 |  |
| 20 | 343 | B | Chronic EBV Infection | 3.19E+05 | 0.00118 |  |
| 21 | 344 | NK | EBV-associated Hemophagocytic Lymphohistiocytosis | 4.08E+05 | 0.00784 | 1.40034 |
| **Table S3. (*continued*)** | | | | | | |
| **Sample No.** | **Patient No.** | **Cell type of samples** | **Diagnosis** | **EBV copies / 2x10^^5^ cells** | **miRNA relative expression** | **DDX3X relative expression** |
| 22 | 346 | T | Chronic Active EBV Infection | 2.72E+07 | 0.00539 |  |
| 23 | 351 | T | Angioimmunoblastic Lymphoma | 6.12E+06 | 0.08260 |  |
| 24 | 365 | B | Aggressive Natural Killer Cell Leukemia | 2.94E+06 | 0.06432 |  |
| 25 | 365 | NK | Aggressive Natural Killer Cell Leukemia | 2.21E+07 | 0.23395 | 4.45847 |
| 26 | 369 | B | Chronic EBV Infection | 1.64E+06 | 0.02838 |  |
| 27 | 372 | NK | Chronic Active EBV Infection | 1.37E+05 | 0.00169 | 3.21551 |
| 28 | 372 | T | Chronic Active EBV Infection | 3.37E+05 | 0.01891 |  |
| 29 | 380 | NK | NK/T-cell Lymphoma | 6.98E+07 | 0.03575 | 4.67432 |
| 30 | 385 | NK | Aggressive Natural Killer Cell Leukemia | 8.14E+06 | 0.73674 | 3.25168 |
| 31 | 391 | NK | NK/T-cell Lymphoma | 1.96E+07 | 0.77117 | 2.65810 |
| 32 | 403 | NK | Hydroa vacciniforme-like lymphoproliferative disorder | 7.36E+06 | 0.19745 | 9.40433 |
| 33 | 417 | NK | Chronic Active EBV Infection | 7.71E+06 | 0.48722 | 4.68628 |
| 34 | 421 | NK | ALK-negative Anaplastic Large Cell Lymphoma | 4.42E+05 | 0.00578 | 4.37333 |
| 35 | 431 | NK | Chronic Active EBV Infection | 1.14E+07 | 0.49212 | 0.58140 |
| 36 | 462 | NK | Extranodal NK/T-cell Lymphoma | 1.30E+08 | 0.60585 | 2.81861 |
| 37 | 472 | NK | Chronic Active EBV Infection | 8.05E+06 | 0.46765 | 2.82034 |
| 38 | 493 | B | Chronic Active EBV Infection | 2.31E+06 | 0.00922 |  |
| 39 | 493 | T | Chronic Active EBV Infection | 4.38E+06 | 0.03313 |  |
| 41 | 495 | T | Chronic Active EBV Infection | 4.39E+07 | 0.67474 | 8.20801 |
| 40 | 495 | NK | Chronic Active EBV Infection | 3.64E+07 | 0.00672 |  |
| 42 | 506 | NK | Chronic Active EBV Infection | 2.40E+07 | 0.23443 | 6.86886 |
| **Table S3. (*continued*)** | | | | | | |
| **Sample No.** | **Patient No.** | **Cell type of samples** | **Diagnosis** | **EBV copies / 2x10^^5^ cells** | **miRNA relative expression** | **DDX3X relative expression** |
| 43 | 511 | NK | Chronic Active EBV Infection | 2.27E+07 | 0.76904 | 3.44007 |
| 44 | 525 | B | Autoimmune Hemolytic Anemia | 2.91E+07 | 0.04366 |  |
| 45 | 529 | B | Chronic Active EBV Infection/Mosquito Bite Disease | 2.22E+06 | 0.02397 |  |
| 46 | 529 | T | Chronic Active EBV Infection/Mosquito Bite Disease | 5.27E+06 | 0.09891 |  |
| 47 | 539 | NK | EBV-associated Hemophagocytic Lymphohistiocytosis | 1.01E+05 | 0.02126 | 4.58517 |
| 48 | 565 | NK | Chronic Active EBV Infection | 1.90E+07 | 0.92899 | 2.23990 |
| 49 | 588 | NK | Chronic Active EBV Infection | 9.17E+05 | 0.12883 | 6.35422 |
| 50 | 601 | B | Angioimmunoblastic Lymphoma | 3.77E+06 | 0.04164 |  |

**SUPPLEMENTARY FIGURES**

**Figure S1. Correlation analysis between EBV-miR expression and DDX3X levels**


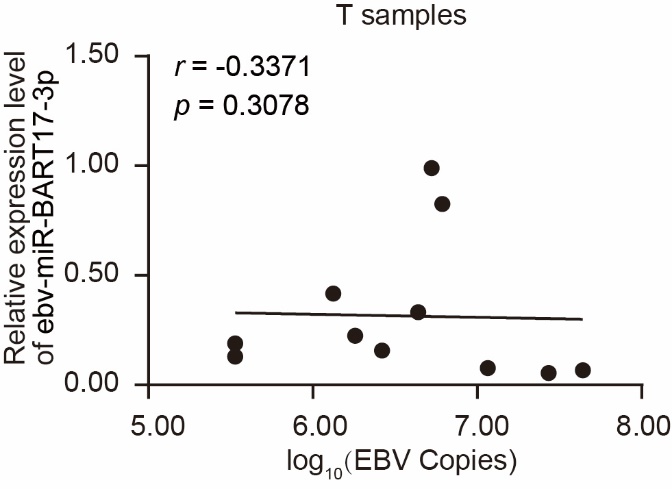


**Figure S1. Correlation analysis between EBV-miR expression and DDX3X levels.** Correlation analysis between EBV-miR-BART17-3p expression and DDX3X levels in T-cell samples (*r* = -0.3371, *P* = 0.3078).
